# Can Electronic Health Records Databases Complement Spontaneous Reporting System Databases? A Historical-Reconstruction of the Association of Rofecoxib and Acute Myocardial Infarction

**DOI:** 10.3389/fphar.2018.00594

**Published:** 2018-06-06

**Authors:** Vaishali K. Patadia, Martijn J. Schuemie, Preciosa M. Coloma, Ron Herings, Johan van der Lei, Miriam Sturkenboom, Gianluca Trifirò

**Affiliations:** ^1^Department of Medical Informatics, Erasmus University Medical Center, Rotterdam, Netherlands; ^2^Sanofi, Bridgewater, NJ, United States; ^3^PHARMO Institute, Utrecht, Netherlands; ^4^Julius Global Health, University Medical Center Utrecht, Utrecht, Netherlands; ^5^Department of Biomedical and Dental Sciences and Morphofunctional Imaging, University of Messina, Messina, Italy

**Keywords:** EU-ADR, WHO-VigiBase, signal detection, EHR, LGPS

## Abstract

**Background:** Several initiatives have assessed if mining electronic health records (EHRs) may accelerate the process of drug safety signal detection. In Europe, Exploring and Understanding Adverse Drug Reactions (EU-ADR) Project Focused on utilizing clinical data from EHRs of over 30 million patients from several European countries. Rofecoxib is a prescription COX-2 selective Non-Steroidal Anti-Inflammatory Drugs (NSAID) approved in 1999. In September 2004, the manufacturer withdrew rofecoxib from the market because of safety concerns. In this study, we investigated if the signal concerning rofecoxib and acute myocardial infarction (AMI) could have been identified in EHR database (EU-ADR project) earlier than spontaneous reporting system (SRS), and in advance of rofecoxib withdrawal.

**Methods:** Data from the EU-ADR project and WHO-VigiBase (for SRS) were used for the analysis. Signals were identified when respective statistics exceeded defined thresholds. The SRS analyses was conducted two ways- based on the date the AMI events with rofecoxib as a suspect medication were entered into the database and also the date that the AMI event occurred with exposure to rofecoxib.

**Results:** Within the databases participating in EU-ADR it was possible to identify a strong signal concerning rofecoxib and AMI since Q3 2000 [RR LGPS = 4.5 (95% CI: 2.84–6.72)] and peaked to 4.8 in Q4 2000. In WHO-VigiBase, for AMI term grouping, the EB05 threshold of 2 was crossed in the Q4 2004 (EB05 = 2.94). Since then, the EB05 value increased consistently and peaked in Q3 2006 (EB05 = 48.3) and then again in Q2 2008 (EB05 = 48.5). About 93% (2260 out of 2422) of AMIs reported in WHO-VigiBase database actually occurred *prior* to the product withdrawal, however, they were reported *after* the risk minimization/risk communication efforts.

**Conclusion:** In this study, EU-EHR databases were able to detect the AMI signal 4 years prior to the SRS database. We believe that for events that are consistently documented in EHR databases, such as serious events or events requiring in-patient medical intervention or hospitalization, the signal detection exercise in EHR would be beneficial for newly introduced medicinal products on the market, in addition to the SRS data.

## Introduction

Rofecoxib is a prescription COX-2 selective Non-Steroidal Anti-Inflammatory Drugs (NSAID) for relief of osteoarthritis signs and symptoms, management of acute pain in adults and treatment of menstrual pain. The European Medicines Agency (EMA) and the United States Food and Drug Administration (FDA) provided approval for rofecoxib in 1999. In June 2000, Vioxx Gastrointestinal Outcome studies (VIGOR) was submitted to FDA which demonstrated an increased risk of cardiovascular thrombotic events, mostly driven by heart attacks (0.5 vs. 0.1% for rofecoxib and naproxen, respectively) (Food and Drug Administration, 2017). However, this finding was initially surprisingly attributed to cardio-protective effect of naproxen. In April 2002, this and other cumulated evidence on potential risks associated to rofecoxib led to the introduction of warnings on rofecoxib labeling concerning the increased risk of cardiovascular events (heart attack and stroke) (Ball et al., 2016; Food and Drug Administration, 2016). Subsequently, in September 2004, the APPROVe study showed increased risk of myocardial infarction and stroke for the 12.5 and 25 mg dose as compared to placebo after 18 months of treatment (Food and Drug Administration, 2017). The same month, the manufacturer withdrew rofecoxib from the market because of concerns about increased risk of heart attack and stroke associated with long-term, high-dosage use (Ritter et al., 2009; Food and Drug Administration, 2017). Subsequent to rofecoxib finding, there has been extensive research done on NSAIDs and cardiovascular risks.

This case triggered dialog in the scientific community about how to improve the post-marketing surveillance of medicines with the aim of achieving early signal detection and ultimately regulatory intervention to ensure patients’ safety (Ritter et al., 2009). In particular, several initiatives have assessed if mining electronic health records (EHRs) may accelerate the process of drug safety signal detection and strengthening. In the United States (US), in 2008, FDA has created the Sentinel System which is a national electronic system for medicinal product safety surveillance (Sentinel, 2017). As of 2016, the Sentinel Distributed Database contained medical and pharmacy benefits data on 178 million members (Sentinel, 2017). Also in 2008, in the United States, a public-private initiative called formerly Observational Medical Outcomes Partnership (OMOP) and currently Observational Health Data Sciences and Informatics (OHDSI) was established to research and educate stakeholders on the appropriate use of EHR for studying the effects of medicines (OMOP, 2017).

In Europe, Exploring and Understanding Adverse Drug Reactions (EU-ADR) Project Focused on using clinical data from EHRs of over 30 million patients from several European countries (Netherlands, Denmark, United Kingdom, and Italy) during 2008–2012 (Coloma et al., 2011). The EU-ADR analyses has showed that signal detection using EHR could complement spontaneous reports that remain the cornerstone of drug safety signal detection, particularly with events occurring at high frequency in the general population and those that are perceived as unlikely to be drug induced (Pacurariu et al., 2015; Patadia et al., 2015a). A retrospective study of EHR in the EU-ADR project demonstrated the value of using EHR data in signal detection and strengthening (Pacurariu et al., 2015; Patadia et al., 2015b).

In the current study we aimed to explore if the signal concerning rofecoxib and acute myocardial infarction (AMI) could have been identified in the EU-ADR distributed healthcare database project earlier than the spontaneous reporting system (SRS) and contribution of EU-ADR data in signal strengthening and possibly earlier rofecoxib withdrawal.

## Materials and Methods

### Data Sources

For this study, data from the EU-ADR project and the World Health Organization’s VigiBase^TM^ (WHO-VigiBase) which is an international spontaneous reporting database were used for the analysis. The EU-ADR project comprised seven established European healthcare databases located in four countries. Health-Search (HSD; Italy), Integrated Primary Care Information (IPCI; Netherlands), and Pedianet (Italy) are primary care databases, where both clinical information including medical diagnoses and drug prescriptions are recorded by general practitioners (IPCI and HSD) or family pediatrician (Pedianet) distributed all over the respective countries. The Aarhus University Hospital Database (Aarhus, Denmark), PHARMO (Netherlands), and the regional Italian databases of Lombardy and Tuscany are comprehensive record-linkage systems in which drug dispensing data of well-defined populations is linked to a registry of hospital discharge diagnoses and other registries collecting clinical information. The main characteristics of the EU-ADR project have been described in more detail by Coloma et al. (2011, 2012). The data collected between the years 1995–2010 were used in this study (Coloma et al., 2012).

For the SRS analysis, the WHO-VigiBase database was used. This database consists of reports of suspected adverse drug reactions (ADRs) received since 1968 from more than 100 member countries. At the time of this analysis, it contained over nine million reports of ADRs worldwide till 2010 (WHO, 2016). The reports originate from various sources including healthcare professionals, consumers, and pharmaceutical manufacturers. The suspected ADRs are coded using the Medical Dictionary for Regulatory Activities (MedDRA) and patient narratives (event history and details) are not included in the public version.

### AMI Search Criteria

Due to the large heterogeneity in event coding between EHR databases in the EU-ADR data, harmonization of event definition was required. The Unified Medical Language System^®^ (UMLS^®^) concepts and related codes and labels corresponding to AMI were identified, and using these codes and terms, database owners constructed their queries for the data extraction. The queries for the event data extraction from different EHR databases were analyzed by a team of clinical experts and, where necessary, were harmonized across all EHR databases. The detailed process is described by Avillach et al. (2010, 2013).

For the analyses in the WHO-VigiBase databases, the MedDRA dictionary including Standardized MedDRA Queries [version 11.1] was reviewed to define custom grouping of terms for AMI which included the following preferred terms: ‘Acute myocardial infarction,’ ‘ECG signs of myocardial ischemia,’ ‘Silent myocardial infarction.’

### Rofecoxib-AMI Association Evaluation

In the EU-ADR project, the Longitudinal Gamma Poisson Shrinker (LGPS), the posterior expectation of the incidence rate ratio [Relative Risk (RR)-LGPS] was developed and estimated for drug-event pair. A RR-LGPS ≥ 2 (*p*-value < 0.05) was classified as a signal, except when the “Longitudinal Evaluation of Observational Profiles of Adverse events Related to Drugs” (LEOPARD) method identified such an association as potentially due to protopathic bias (Gerhard, 2008; Schuemie, 2011; Schuemie et al., 2012). The relative risks of AMI during exposure to rofecoxib as compared to non-exposure to the drug was calculated on quarter of year basis by measuring LGPS values, WHO-VigiBase analyses were conducted on data up to and including 4th quarter of 2010 using Oracle Empirica^TM^ Signal (Waltham, MA, United States). The Gamma Poisson Shrinker (GPS) was used to compute EB05 (Empirical Bayes posterior Gamma Mixture 5th percentile; estimates lower point in 90% confidence interval). A threshold of EB05 ≥ 2 (*p*-value < 0.05) was selected based on extensive use and validation in PV practice (Szarfman et al., 2002). The analyses were conducted two ways- based on the date the AMI events with rofecoxib as a suspect medication were entered into the WHO-VigiBase database and also the date that the AMI event occurred with respect to the exposure to rofecoxib. The data mining quarter date was used as a surrogate for the event date.

## Results

### EU-ADR Analysis

**FIGURE 1:**
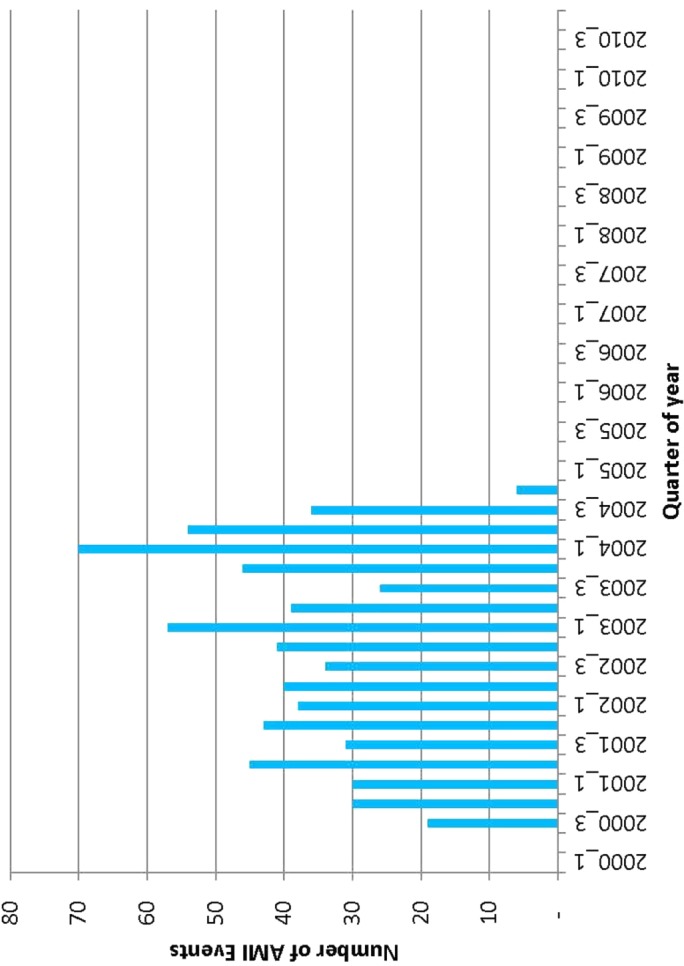
Frequency of acute myocardial infarction (AMI) events occurring during exposure to rofecoxib in the EU-ADR database network.

A total of 685 AMI events during exposure to rofecoxib were captured in the databases participating in the EU-ADR project during the years 2000–2010 (**Figure 1**). The first AMI event during rofecoxib was recorded in third quarter of the year 2000 with total of 49 AMIs for the rest of that year. After withdrawal of rofecoxib there have not been any new AMI events during exposure, since 2005.

**FIGURE 2:**
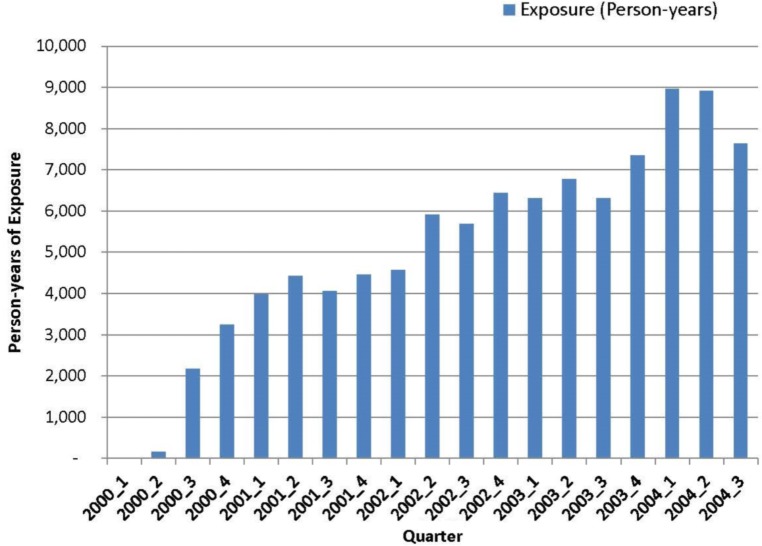
Rofecoxib exposure (person-years) cumulated over time in the EU-ADR database network.

The rofecoxib market penetration in person-years of exposure in the participating EU databases is shared in **Figure 2**. Rofecoxib uptake began in the second quarter of the year 2000 with approximately 5,562 person-years of exposure in the year 2000. Its quarter-by-quarter exposure peaked in the first quarter of 2004 with 8,959 person-years of exposure. Subsequent to that, the exposure rapidly declined throughout 2004.

**FIGURE 3:**
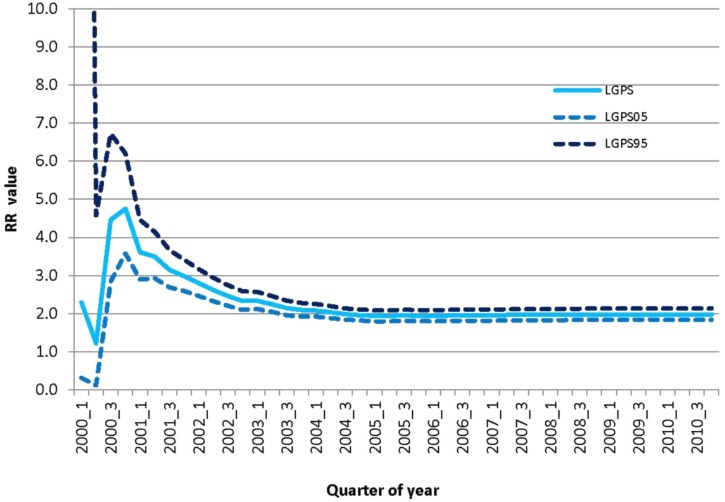
Relative risk of AMI associated to rofecoxib use vs. non-use over time in the EU-ADR database network. Relative risk (RR) was measured as Longitudinal Gamma Poission Shrinkage (LGPS) value together with 95% confidence interval.

The databases in the EU-ADR project were able to identify a strong association concerning rofecoxib and AMI since the third quarter of 2000 (RR LGPS = 4.5; 95% Confidence Interval: 2.84–6.72) (see **Figure 3**). The threshold of RR LGPS > = 2 was crossed early in 2000. The RR LGPS value increased to 4.5 in the third quarter of 2000 and peaked to 4.8 in the fourth quarter of 2000. The RR LGPS value ranged between 3 and 4 in the year 2001 and between 2 and 3 in the year 2002. It subsequently stabilized around 2 and stayed above the threshold of 2 until 2005.

### WHO-VigiBase Analysis

**FIGURE 4:**
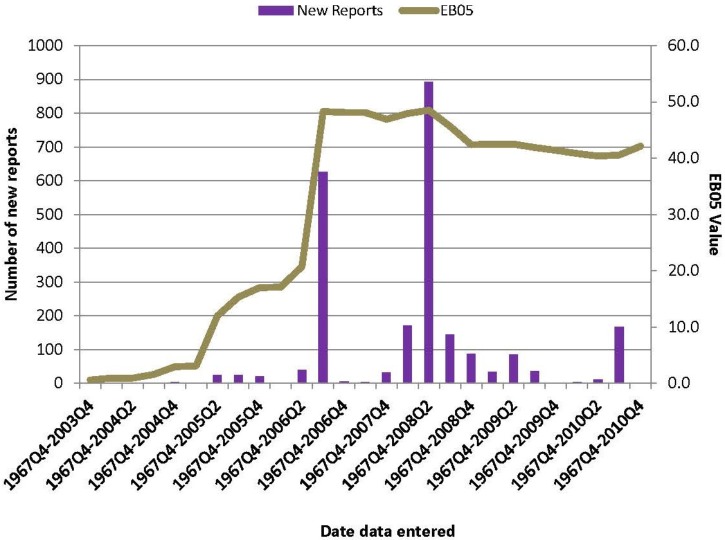
Distribution of reports of AMI for which rofecoxib was the suspected drug as collected over time in the WHO- VigiBase spontaneous reporting database. EB05: Empirical Bayes posterior Gamma Mixture 5th percentile; estimates lower point in 90% confidence interval.

In the WHO-VigiBase SRS database, a total of 2,422 reports of AMIs were received with rofecoxib as a suspect medication. **Figure 4** shows the WHO-VigiBase data on rofecoxib and AMI per quarter by the date data were reported and entered in the WHO-VigiBase database. The first report of AMI with rofecoxib as a suspect medication was submitted in the fourth quarter of 2003, after the initial warning in 2002. There were two large increases in the number of new reports that were observed in the third quarter of 2006 and the second quarter of 2008. The EB05 threshold of 2 was crossed in the fourth quarter of 2004 (EB05 = 2.94). Since then, the EB05 value increased consistently and peaked in the third quarter of 2006 (EB05 = 48.3) and then again in second quarter of 2008 (EB05 = 48.5). It declined slightly after 2008 but still stayed in the range of 40–45.

**FIGURE 5:**
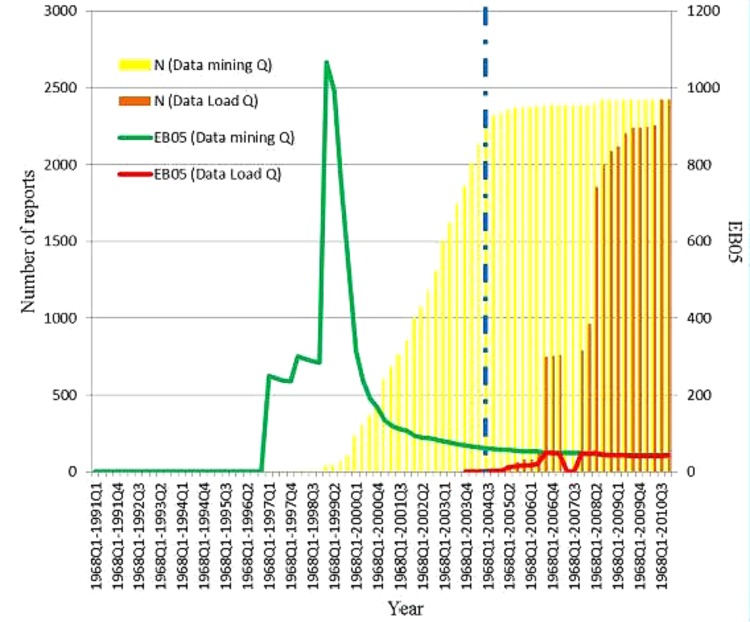
Acute myocardial infarction and Rofecoxib in WHO-VigiBase database. The yellow bars in the figure show the number of cumulative reports by quarter using the data mining quarter date. The orange bats represent the cumulative reports by the date they were reported and entered into WHO-VigiBase. The vertical blue line marks the date the manufacturer withdraw the product. The EBOS values using the data mining quarter is shown in greenline and using the data load quarter is shown in red line.

**Figure 5** shows the WHO-VigiBase data on rofecoxib and AMI per quarter by the date data were entered in the WHO-VigiBase database (data load date) and also the date that the actual event occurred (data mining quarter date). The data from this chart shows that 93% (2,260 out of 2,422) of acute myocardial events actually occurred *prior* to the product withdrawal. However, they were reported *after* the risk minimization/risk communication efforts.

## Discussion

We demonstrated in this study that the AMI signal concerning rofecoxib could have been detected in the year 2000 (or 2001 at latest, taking into account the lag time in getting access to large database network) using the EU-ADR network of claims databases and EMRs, about 4 years before the signal was identified in the SRS data. The signal was not identified earlier in the WHO-VigiBase SRS. This is noteworthy since the association of AMI and rofecoxib was already documented in the VIGOR trial in which the finding on threefold increased cardiotoxicity in rofecoxib users vs. naproxen users was misinterpreted as protective effect of the latter drug (Juni et al., 2004).

For the past six decades, for the marketed medicinal products, using spontaneously reported adverse events data has been the “gold standard” in the pharmacovigilance practice, even though, the limitations of these data are well-recognized, including under-reporting in general and also over-reporting of highly publicized drug-adverse effects.

Healthcare professionals may not properly and readily attribute the onset of a multifactorial event like AMI to a medicine especially if that medicine is used for treating a disease which is *per se* a strong cardiovascular risk factor as it is the case for rheumatology diseases requiring coxib treatments. In the WHO-VigiBase database, initially, extremely low number of reports of suspected rofecoxib-associated AMI was reported. As a paradox, in the years following the rofecoxib withdrawal, a huge increase in the number of reports was observed. Data show that 93% of the reports concerned AMIs that occurred *prior* to the initial risk communications, however, was reported only after the rofecoxib was withdrawn from the market. These data show that the publicity of this topic was the driving force behind the increase number of reports observed *after* a risk was identified and not the true increase in the incidence of the drug-event combination.

It is possible, that once the risk was confirmed, communicated, and action taken by a regulatory agency, possibly healthcare professionals felt supported or even validated, in some instances, to report. In some cases, perhaps healthcare professional were even feeling compelled to report once a risk communication was distributed. Another major phenomenon to consider is that legal actions are common in North America, once a risk is communicated to public. Is the ‘encouragement’ from the lawyers driving patients and/or health care professionals to recall and report AMIs retrospectively? It will be interesting to tease out these reasons in future research.

Electronic health record data are more immune to this type of reporting bias. The data are collected as a by product of the healthcare delivery practice and medical records system. They are not dependent on a healthcare professional or patient to, first, identify such an event and then report the event. The collection of the events and outcomes is less or not at all influenced by media or legal actions. Although not all events and outcomes are consistently captured in the EHR databases, serious events, such as AMI, have a greater chance to be collected and accurately coded.

As mentioned earlier, the SRS databases are used as a “gold-standard” in the pharmacovigilance practice for marketed products. However, the increased number of ADRs in the SRS after identification and communication of a risk brings minimal value to pharmacovigilance scientists. In fact, they contribute to “noise” in the SRS database. It is important for the pharmacovigilance scientists to understand this phenomenon when they are conducting data mining and signal detection for same medical concept but in different marketed product.

It is important to note limitations of this study. First, the study focused only on AMI as adverse event associated to with rofecoxib. As a consequence, the findings may not be generalizable to a broader range of drug-event associations, especially non-serious events which may not be captured consistently in the EHR system or drugs which are used in different therapeutic areas as compared to rofecoxib. In addition, WHO-VigiBase data were used for the SRS analyses. There are several other SRS databases that are widely used in the pharmacovigilance practice. The results may not be generalizable to other SRS databases. Another limitation is the fact that the LGPS method used on EHR data does not correct for confounding (Lawrence Gould, 2014), and the increase observed might therefore reflect bias rather than a true signal. Lastly, true timing of the detection of AMI-rofecoxib association has to take into account the lag time between data generation and data access which may delay of 6 months or even 1 year the association identification in prospective evaluation. Future research should focus on these issues.

## Conclusion

In this EU database network study covering a source population of 30 million persons, we were able to detect the AMI-rofecoxib association around 4 years prior to the drug withdrawal. If such a network was in place at that time it may have theoretically speed up the process leading to rofecoxib removal from the market. More specifically, the AMI-rofecoxib signal was initially identified in RCT but misinterpreted. As Platt stated in the Institute of Medicine meetings, large EHR and claims database networks may complement SRS and other sources for post-marketing drug safety evaluation, especially for those adverse events which are frequently captured in EHR databases and are not likely to be reported to SRS (Patadia et al., 2015a). The United States implemented Sentinel and the Canadian Network for Observational Drug Effect Studies (CNODES) has expanded capacity for drug safety surveillance. The EU has implemented several projects; however, none with sustainable system yet exist.

## Author Contributions

The EU-ADR database analyses were conducted by MJS, PC, and GT, under the guidance of MS. The WHO-VigiBase analyses along with manuscript writing were done by VP. GT provided significant guidance to VP with manuscript development. MS, JvdL, and RH provided editorial review and research mentorship. All authors provided critical revision of the manuscript for important intellectual content.

## Conflict of Interest Statement

VP is an employee of Sanofi, the work described here is part of her Ph.D. research and outside of her scope of work at Sanofi. MJS has become a full-time employee of Janssen R&D since finishing this research. PC has become a full-time employee of F. Hoffmann-La Roche since this research was completed. RH is an employee of the PHARMO Institute. This independent research institute performs financially supported studies for government and related healthcare authorities and several pharmaceutical companies. MS was leading a research group in Erasmus MC that was conducting research for pharmaceutical companies, including Novartis, Servier, GSK, J&J. The remaining authors declare that the research was conducted in the absence of any commercial or financial relationships that could be construed as a potential conflict of interest.
